# Overall water splitting by Pt/g-C_3_N_4_ photocatalysts without using sacrificial agents†
†Electronic supplementary information (ESI) available: Characterization and experimental detail. See DOI: 10.1039/c5sc04572j


**DOI:** 10.1039/c5sc04572j

**Published:** 2016-01-27

**Authors:** Guigang Zhang, Zhi-An Lan, Lihua Lin, Sen Lin, Xinchen Wang

**Affiliations:** a State Key Laboratory of Photocatalysis on Energy and Environment , College of Chemistry , Fuzhou University , Fuzhou , 350002 , China . Email: xcwang@fzu.edu.cn ; http://www.wanglab.fzu.edu.cn

## Abstract

We report the direct splitting of pure water by light-excited graphitic carbon nitride (g-C_3_N_4_) modified with Pt, PtO_*x*_, and CoO_*x*_ as redox cocatalysts, while pure g-C_3_N_4_ is virtually inactive for overall water splitting by photocatalysis. The novelty is in the selective creation of both H_2_ and O_2_ cocatalysts on surface active sites of g-C_3_N_4_*via* photodeposition triggering the splitting of water for the simultaneous evolution of H_2_ and O_2_ gases in a stoichiometric ratio of 2 : 1, irradiated with light, without using any sacrificial reagents. The photocatalyst was stable for 510 hours of reaction.

## Abstract 2

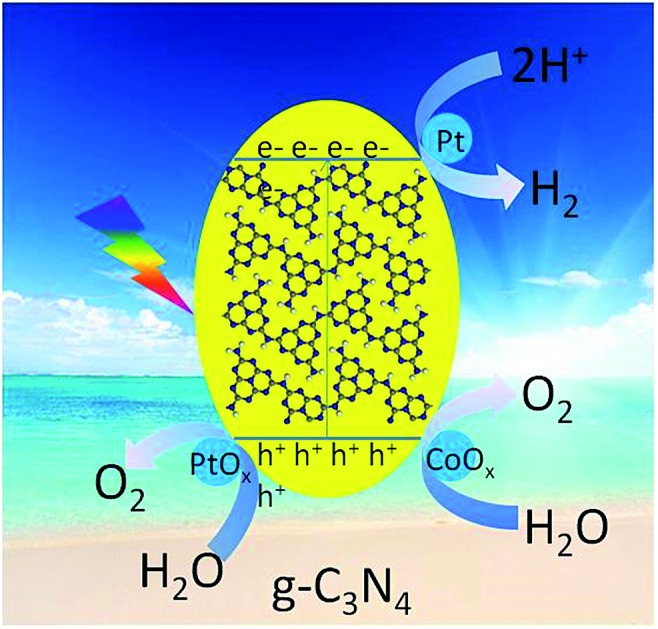
Direct splitting of pure water into H_2_ and O_2_ in a stoichiometric molar ratio of 2 : 1 by conjugated polymers *via* a 4-electron pathway was established for the first time, as demonstrated here using a g-C_3_N_4_ polymer and redox co-catalysts of Pt and Co species.

## 

Using photocatalysts to produce hydrogen sustainably by water splitting is the “holy grail” in modern science. Over the past 40 years, inorganic semiconductors, such as metal oxides and metal (oxy)nitrides, have been utilized as photocatalysts for hydrogen production.^1^–^8^ However, direct water splitting in a wireless powder photocatalytic system to produce gaseous hydrogen and oxygen has not yet been achieved using conjugated polymers (CPs). These materials have already shown great promise for use in organic electronics and photovoltaic devices, such as solar cells, light-emitting diodes, and field-effect transistors, due to their good processability and tuneable electronic structures.^9^–^13^

The key challenge to using pristine CPs for direct water splitting is the insufficient hopping charge transport of the chains (usually below 10^–4^ cm^2^ V^–1^ s^–1^) and a poor stability in water and under light irradiation.^12^ Increasing the structural dimensions of the CPs (*e.g.*, from 1D chains to 2D architectures) is desirable because the hole mobility is greatly increased (up to 0.1 cm^2^ V^–1^ s^–1^) by the remarkably reduced binding energies of the Frenkel-type excitons and the robust stability of the 2D extended π-conjugated units.^14^ However, further progress in direct water splitting by CPs will rely on breakthroughs in combining stable CP light transducers with suitable redox cocatalysts (usually noble metals) to promote charge separation and to reduce charge build-up on the polymer surface to prevent photocorrosion. Indeed, the promise of this type of system has been demonstrated by the successful development of 2D graphitic carbon nitride (g-C_3_N_4_) polymer and metal-based redox cocatalyst systems for CO_2_ reduction, organic synthesis and water half-splitting reactions using sacrificial reagents.^15^–^22^ In contrast, it is difficult to achieve overall water splitting without using sacrificial reagents because it depends not only on a rational chemical synthesis to tune the textural properties of the polymer but also on a rational design of the composite to control the reaction kinetics on the polymer surface.^23^–^27^

Photocatalytic water splitting by a prototypical g-C_3_N_4_ polymer was shown to be thermodynamically possible because the C_2p_ and N_2p_ orbital bands straddle the water splitting redox potentials,^15^–^22^,^28^–^34^ but pure g-C_3_N_4_ is typically limited by sluggish kinetics in photocatalyzing overall water splitting due to a lack of surface redox active sites. By optimizing the g-C_3_N_4_ bulk and morphological properties and employing suitable redox cocatalysts (*e.g.*, Pt for H_2_ evolution and Co(OH)_2_ for oxygen evolution), activities for the water half-splitting reactions (water reduction and oxidation) can be dramatically increased.^28^–^34^ Therefore, if the appropriate water redox cocatalysts are simultaneously deposited on g-C_3_N_4_, pure water splitting to produce gaseous hydrogen and oxygen could be achieved. However, the rough deposition of cocatalysts by traditional chemical reduction (*e.g.*, H_2_ and NaBH_4_) cannot fully amplify the activity. Besides, the densely stacked graphitic layer also causes trouble for charge separation and migration due to a long bulk diffusion distance, resulting in a low photocatalytic quantum efficiency.^15^ It is advisable to reduce the diffusion distance by rational synthesis of a g-C_3_N_4_ nanosheet together with suitable cocatalyst modification to achieve water splitting. Up to now, direct water splitting photocatayzed by g-C_3_N_4_ CPs in the absence of sacrificial reagents has never been realized and still remains a significant basic science challenge. Here, we demonstrate that light-excited g-C_3_N_4_ CPs can induce a one-step water splitting reaction *via* a four-electron pathway to generate gaseous H_2_ and O_2_ in a stoichiometric molar ratio of 2 : 1 when their morphology is modified and the reaction kinetics are improved by modification with Pt, PtO_*x*_, and CoO_*x*_*via* photodeposition. The optimal g-C_3_N_4_-based nanocomposite had a turnover number of 3.1 moles of H_2_ and O_2_ per mole of g-C_3_N_4_ photocatalyst for the overall water splitting reaction. The nanocomposite was stable in water and under light irradiation.

The g-C_3_N_4_ polymers used for photocatalytic water splitting were typically prepared by thermally polymerizing urea into heptazine units at 550 °C which pack together like graphitic crystals. This structure was confirmed by X-ray diffraction (XRD), Fourier transform infrared (FT-IR) spectroscopy, and Raman spectroscopy (Fig. S1†).^15^,^35^–^37^ The g-C_3_N_4_ optical properties measured by UV-vis diffuse reflection spectroscopy (DRS) were characteristic of a semiconductor; g-C_3_N_4_ had an optical absorption edge at 442 nm due to the excitation of electrons from its valence band to its conduction band (Fig. 1a). The conduction band minimum (CBM) and valence band maximum (VBM) of the g-C_3_N_4_ semiconductor were determined to be –1.31 V and 1.49 V (*vs.* NHE, pH = 7), respectively, from electrochemical Mott–Schottky plots (Fig. 1b and c), where an estimated flat potential was directly used as the conduction band potential. Density functional theory (DFT) calculations revealed that the band gap was 2.56 eV with the CBM and VBM located at –1.0137 and 1.5505 V (*vs.* NHE, pH = 7), respectively, which enables g-C_3_N_4_ to act as a redox shuttle for the water splitting reaction (Fig. S2†). This calculated band gap is consistent with the experimental data and further demonstrates that in theory, g-C_3_N_4_ could be used to split water.

**Fig. 1:**
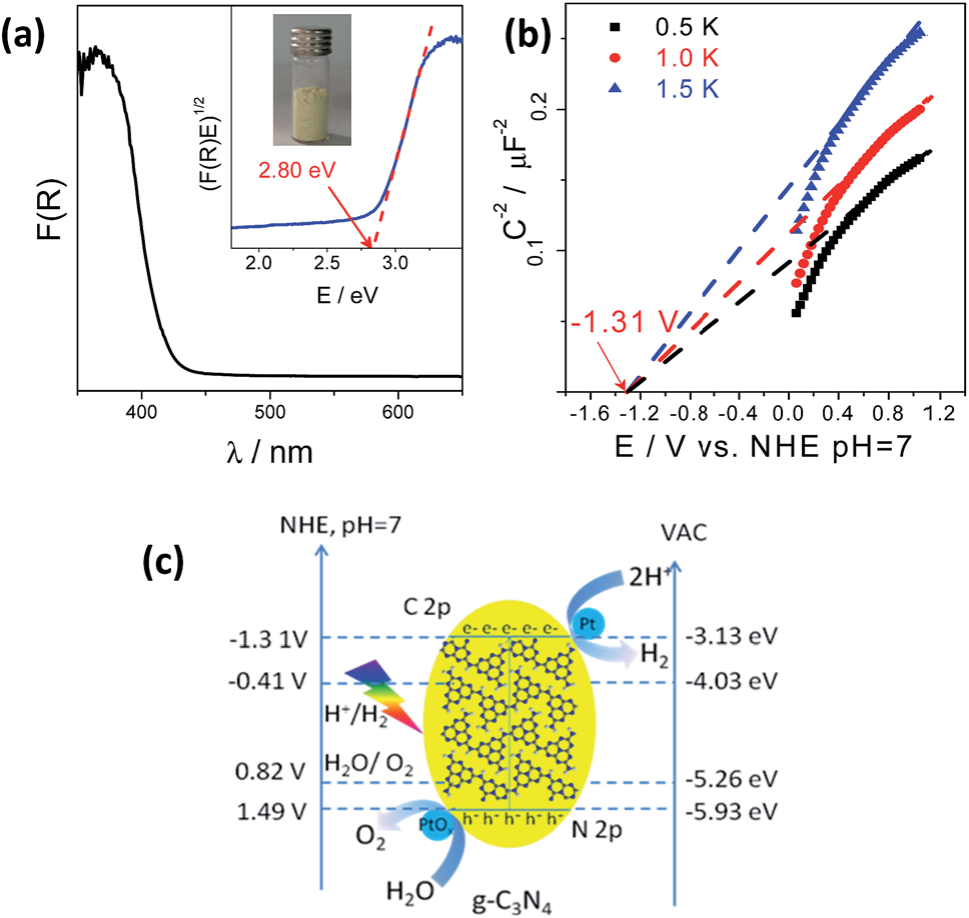
(a) UV-vis DRS spectrum of g-C_3_N_4_ polymers; inset: the corresponding Tauc plot. (b) Mott–Schottky plots of the g-C_3_N_4_ electrode in 0.2 M Na_2_SO_4_, pH = 7. (c) Band structure diagram of g-C_3_N_4_ polymers calculated by optical absorption and typical electrochemical Mott–Schottky methods.

First, the effects of g-C_3_N_4_ morphology on the photocatalytic activity were investigated. We prepared three types of g-C_3_N_4_ using dicyandiamide (DCDA), ammonium thiocyanate (ATC) and urea as precursors. The results showed that after *in situ* photo-deposition with Pt, the urea-derived g-C_3_N_4_ exhibited significant photocatalytic activity for the overall water splitting reaction, while the other samples were inactive for overall water splitting (Table S1†). It should be noted that all pure g-C_3_N_4_ polymers showed no activity for overall water splitting in the absence of cocatalysts, implying that surface kinetic control using Pt species was indispensable to achieve overall water splitting by g-C_3_N_4_ based photocatalysts. N_2_ sorption measurements revealed that the DCDA- and ATC-derived g-C_3_N_4_ samples had smaller surface areas than the urea-derived samples (*ca.* 10 m^2^ g^–1^*vs.* 61 m^2^ g^–1^). However, mpg-C_3_N_4_ with a surface area of *ca.* 67 m^2^ g^–1^ also exhibited no water splitting activity. This indicated that surface area was not the major factor in controlling the water splitting activity and the splitting of water on densely stacked g-C_3_N_4_ polymers was indeed very difficult to achieve. To better understand the real mechanism of water splitting on the soft surface of the CPs, we characterized the morphology of the above different polymers. TEM images of DCDA- and ATC-derived g-C_3_N_4_ and mpg-C_3_N_4_ samples revealed densely stacked polymer layers, which were very different from the silk-like thin nanosheets of the urea-derived one (Fig. S3†). The fast evolution of O in the form of CO_2_ or CO could accelerate the deamination rate. Thus, the texture, morphology and electronic properties of the CNU samples were optimized, and contributed to creating the active Pt/g-C_3_N_4_ photocatalysts for overall water splitting. Evidently, accelerated charge separation and migration on the nanosheets can be obtained in comparison with densely stacked graphitic layers, which is elucidated well by the corresponding large decrease of PL emission intensity (Fig. S4†). The nanosheet structure can also be certified by an AFM experiment. As shown in Fig. 2a, the thickness of the nanosheet is determined as ∼2 nm. One can now easily conclude that the ultrathin 2D geometry of urea-derived g-C_3_N_4_ is crucial for achieving overall water splitting as demonstrated by the fact that g-C_3_N_4_ samples prepared from urea at different temperatures all have remarkable water splitting activities (Fig. S5†) due to their similar thin nanosheet structures (Fig. S6†). The CNU samples prepared at 550 °C showed optimum activities. This is because when the temperature is lower than 550 °C, the heptazine cycle doesn't completely form, while partial decomposition occurs when the temperature is higher than 550 °C. Both of these two aspects may generate inactive CNU samples. DCDA- and ATC-derived g-C_3_N_4_ and mpg-C_3_N_4_ samples revealed densely stacked polymer layers, and the deposition rate of Pt nanoparticles on the surface of the polymer was very slow, in the absence of organic sacrificial agents to react with the holes. Optimization of the deposition technique of Pt is needed to enhance the overall water splitting activities of this bulky g-C_3_N_4_.

**Fig. 2:**
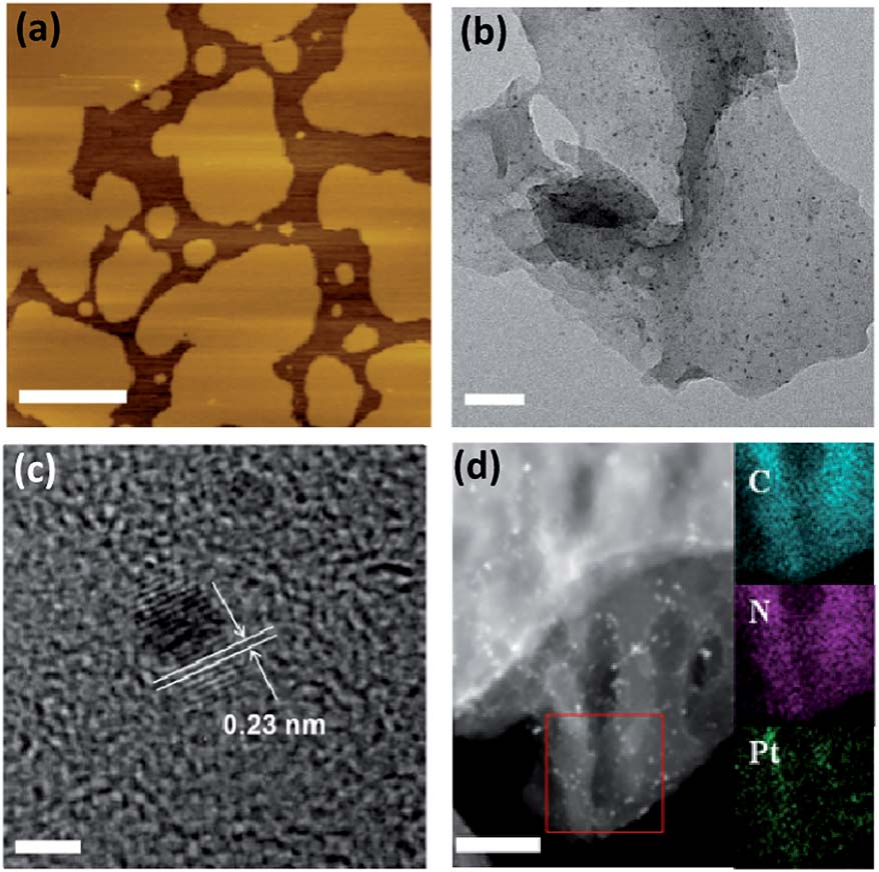
(a) AFM image of the g-C_3_N_4_ polymers with Pt deposited *in situ*. (b) TEM image of the g-C_3_N_4_ polymers with Pt deposited *in situ*. (c) HR-TEM image of the g-C_3_N_4_ polymers with Pt deposited *in situ*. (d) STEM images of the g-C_3_N_4_ polymers with Pt deposited *in situ*. Scale bar for *a*, *b*, *c* and *d* is 50 nm, 100 nm, 2 nm and 50 nm, respectively.

We then investigated the effect of cocatalyst loading techniques on the photocatalytic water splitting activity. Three different cocatalyst loading techniques, *in situ* photodeposition, and H_2_ and NaBH_4_ reduction, were developed to decorate the g-C_3_N_4_ nanosheets. As shown in Fig. S7,† evident water splitting activity was generated for photodeposition of Pt on the surface of the g-C_3_N_4_ nanosheets, while only very slow H_2_ and no O_2_ evolution were found for both H_2_ and NaBH_4_ reduction modified ones. In the first case, when g-C_3_N_4_ was irradiated with light, photoexcited charge carriers were generated and then immediately migrated to the surface of the g-C_3_N_4_ nanosheets without recombination. The surface adsorbed Pt^4+^ was then reduced *in situ* by the excited electrons and deposited on the active sites, which can efficiently promote the water splitting. For the other investigated techniques, Pt^4+^ was reduced by H_2_ or NaBH_4_ and then randomly deposited on the surface, resulting in poor activities. The selective photodeposition of Pt on thin g-C_3_N_4_ nanosheets resulted in a uniform dispersion of ultrafine Pt nanoparticles (∼1–2 nm) with a (111) crystal lattice spacing of ∼0.23 nm (Fig. 2b and c). The homogeneous deposition of Pt can be further proved by STEM imaging (Fig. 2d). However, serious particle accumulation occurred when the Pt cocatalysts were deposited by H_2_ and NaBH_4_ reduction (Fig. S8†), which was the major hindrance which led to decreased water splitting activity.

We also investigated the chemical composition and valence state of the Pt species. As shown in Fig. 3a and b, electron energy loss spectroscopy (EELS) and XRD analysis confirmed the existence of a Pt (111) plane.^38^ Besides, no evident structure variation occurred after modification with the Pt cocatalysts, implying a robust stability of the g-C_3_N_4_ CPs.^39^–^41^ Three pairs of XPS peaks corresponding to Pt^0^, Pt^2+^, and Pt^4+^ with binding energy at 72.13, 74.26 and 78.17 eV, respectively, were measured (Fig. 3c). Pt^0^ was effective for H_2_ evolution while PtO_*x*_ were able to promote O_2_ evolution.^42^ However, two pairs of XPS peaks were deconvoluted for a NaBH_4_ reduction modified one (Fig. S9†), indicating the complete reduction of Pt^4+^ into Pt^2+^ and Pt^0^. To confirm that PtO_*x*_ were active for the promotion of a water oxidation reaction, we evaluated the photocatalytic water oxidation activities of the as-prepared PtO_*x*_/g-C_3_N_4_. As shown in Fig. S10,† this material showed enhanced activity for water oxidation in comparison with the pure one, emphasizing the positive role of PtO_*x*_ in improving the water oxidation rate. In addition, the water splitting rates and evolved H_2_/O_2_ gas ratio (Fig. S11†) could be finely tuned by simply adjusting the total loading from 0.2 to 5 wt% due to the change of the ratio of Pt and PtO_*x*_ intensities (Fig. S12 and Table S2†) and the alteration of particle size (Fig. S13†). The creation of metal/polymer surface junctions promotes the interfacial redox reaction which can be confirmed by a rapidly decreased PL intensity (Fig. 3d). The optimum activity was achieved at a Pt loading content of 3 wt%. When Pt or PtO_*x*_ were singly deposited on the g-C_3_N_4_ nanosheets, the sample exhibited very poor activity in both cases, which once again highlighted that the simultaneous creation of both H_2_ and O_2_ evolution cocatalysts on the active sites was indeed essential for triggering the overall splitting of water.

**Fig. 3:**
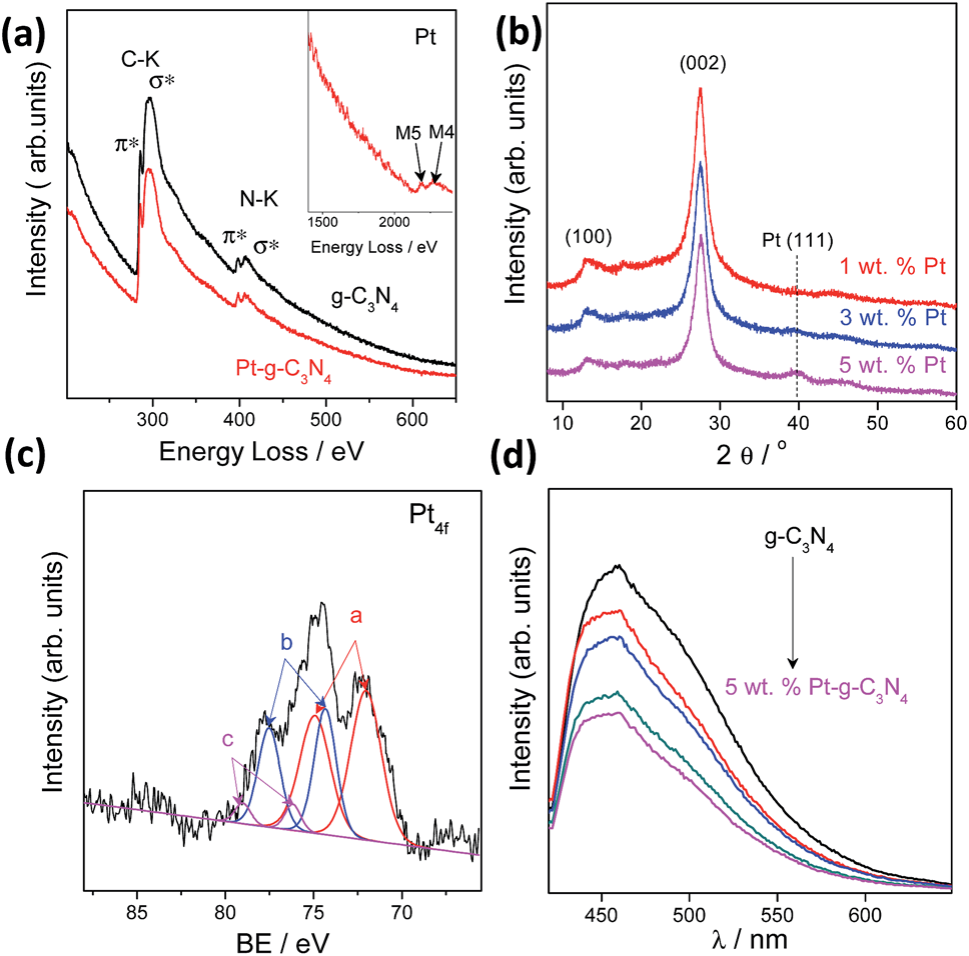
(a) EELS of the g-C_3_N_4_ polymers with Pt deposited *in situ*. (b) XRD of the g-C_3_N_4_ polymers with Pt deposited *in situ*. (c) High resolution of XPS analysis of Pt_4f_. (d) PL spectra of the g-C_3_N_4_ polymers with Pt deposited *in situ*.

The g-C_3_N_4_ nanosheets modified by other noble metals (*e.g.*, Rh, Ru, or Au) *via in situ* photodeposition all just showed trace H_2_ and no O_2_ evolution (Fig. S14†), implying the importance of Pt for water splitting. The pH value and amount of polymer powders used for water splitting were also optimized (Fig. S15 and S16†). The optimum water splitting rate was obtained for samples prepared by photodepositing 3 wt% Pt on 0.2 g of g-C_3_N_4_ nanosheets under neutral conditions. We then evaluated their stability for long term reaction.

As shown in Fig. S17,† the optimized Pt/g-C_3_N_4_ showed good water splitting stabilities under both UV and visible light irradiation for 580 hours of continuous reaction. It should be noted that N_2_ gas was evolved along with H_2_ and O_2_ at the initial stage of the reaction. This arises from the self-oxidation of the surface un-condensed amino groups (–NH) by excited holes.^43^–^45^ As the reaction proceeded, after 80 hours almost no N_2_ evolution was observed, suggesting a complete consumption of the –NH groups. When the Xe lamp was turned off, the amounts of the evolved gases quickly diminished in just four hours (Fig. S18†), indicating a fast occurrence of the backward reaction of water splitting on the Pt species (H_2_ and O_2_ recombination for water formation). Thus, to further enhance the overall water splitting activity of the system, an efficient restraint of the backward reaction *via* rational structural design of the cocatalysts (*e.g.*, core/shell nanostructure) should be considered.

The addition of cobalt species for *in situ* formation of cobalt-based cocatalysts can also sufficiently promote the water oxidation selectivity and efficiency of metal-free semiconductors such as g-C_3_N_4_ and h-BCN.^43^–^47^ As expected, the simultaneous evolution of H_2_ and O_2_ gases in a stoichiometric ratio of 2 : 1 by Pt–Co/g-C_3_N_4_ under UV (*λ* > 300 nm) (12.2 and 6.3 μmol h^–1^) (Fig. 4a) and visible light irradiation (*λ* > 420 nm) (1.2 and 0.6 μmol h^–1^) (Fig. 4b) was significantly enhanced after 1 wt% CoO_*x*_ were further modified for use as O_2_ evolution cocatalysts, which can be determined by XPS analysis (Fig. S19†). The slightly decreased activity in each run of reaction may be attributed to the stacked samples on the inner side of the reactor (Fig. S20†). Furthermore, no obvious deactivation was observed after 510 hours of reaction (Fig. S21†), demonstrating the robust resistance of the composites to water and light corrosion at the soft interface. The total amount of gaseous H_2_ and O_2_ collected reached ∼6.2 mmol, which corresponded to turnover numbers (TON) of 3.1 and 111.3 based on g-C_3_N_4_ and Pt, respectively. The apparent quantum yield (AQY) for the overall water splitting reaction was calculated to be 0.3% at 405 nm (Fig. S22†) and was monitored by an on-line gas chromatograph (Fig. S23†). This is lower than the value of 2.5% of (Ga_1–*x*_Zn_*x*_) (N_1–*x*_O_*x*_) inorganic photocatalysts. However, it is a remarkable first observation that photocatalytic overall water splitting can occur on the surface of an organic/polymer semiconductor *via* a 4-electron pathway. Optimization of the system to further improve the efficiency is ongoing in our lab.

**Fig. 4:**
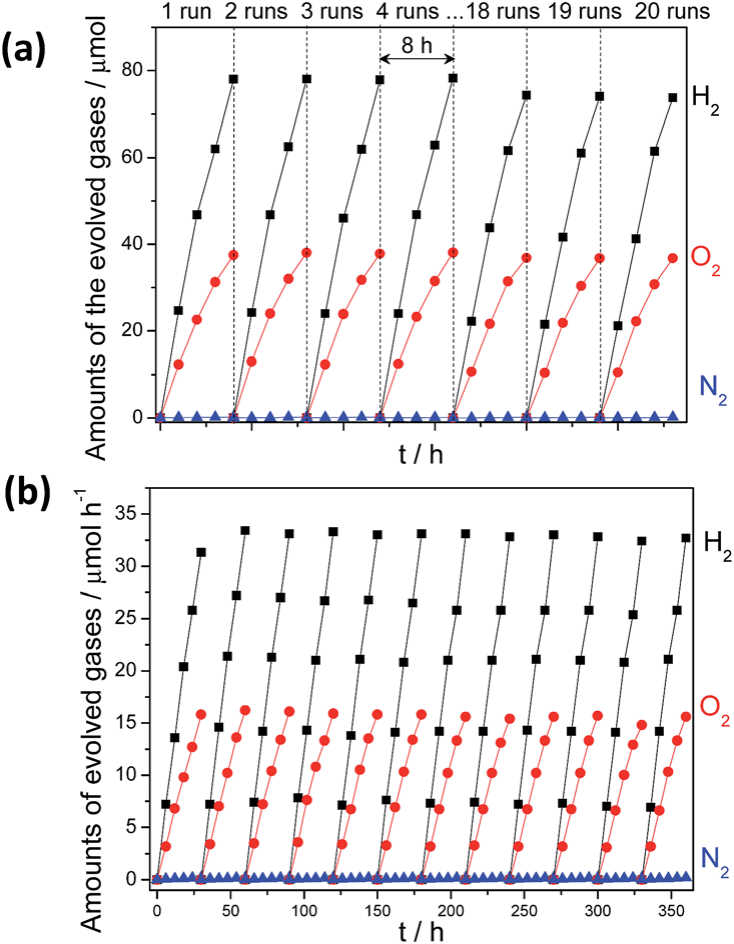
Time course of water splitting activities of 3 wt% Pt, PtO_*x*_ and 1 wt% CoO_*x*_ Co-modified g-C_3_N_4_ polymers under (a) UV-vis (*λ* > 300 nm) irradiation and (b) visible light (*λ* > 420 nm) irradiation.

## Conclusions

The discovery of Pt/g-C_3_N_4_ CPs that can split pure water without the use of sacrificial reagents establishes a new chemical paradigm for exploiting clean, renewable solar energy using organic semiconductor light-energy transducers. Ongoing efforts are focused on modifying the electronic and textural structures of g-C_3_N_4_ CPs and coupling them to low-cost kinetic promoters to facilitate photocatalytic cascade processes for water splitting and CO_2_ fixation that are relevant to sustainable energy production *via* artificial photosynthesis.^48^–^50^

## Supplementary Material

Supplementary information
