# Supplementary material for: Overall water splitting by Pt/g-C3N4 photocatalysts without using sacrificial agents
Source: Chem Sci. 2016 Jan 27;7(5):3062–6. doi: 10.1039/c5sc04572j (PMC6005136; doi:10.1039/c5sc04572j)
Supplement: Supplementary file 1 [file SC-007-C5SC04572J-s001.pdf]

Electronic Supplementary Material (ESI) for Chemical Science.
This journal is © The Royal Society of Chemistry 2016

Supporting Information

Guigang Zhang, Zhi-An Lan, Lihua Lin, Sen Lin and Xinchen Wang*

State Key Laboratory of Photocatalysis on Energy and Environment, College of Chemistry, Fuzhou University, Fuzhou, 350002, China.

E-mail: xcwang@fzu.edu.cn

Methods

Catalysts preparation. g-C_3N_4 was prepared according to the literature. Briefly, 10 g of urea was placed in a crucible with a cover. It was heated in muffle furnace with a heating rate of 5 ^oC/min to the target temperatures (500, 525, 550, 575 and 600 ^oC) and maintained for 2 hours in the air flow. Then the final products were collected after naturally cooled down to the room temperature. In comparison, some other precursors including dicyandiamide (DCDA) and ammonium thiocyanate (AT) were also prepared with the same methods. They were denoted as CND and CNA for simply. Mesoporous carbon nitride (mpg-C_3N_4) was prepared via a classical hard-template strategy. To prepare mpg-C_3N_4, cyanamide (3 g, 72 mmol; Aldrich) was dissolved in 1.5 g of a 40% dispersion of 12-nm SiO_2 particles (Ludox HS40, Aldrich) in water with stirring at 80 ^oC overnight. The resulting transparent mixtures were then heated at a rate of 2.3 ^oC min over 4 h to reach a temperature of 550 ^oC and then tempered at this temperature for another 4 h. The resulting brown-yellow powder was treated with a 4M NH_4HF_2 for 24 h to remove the silica template. The powders were then centrifuged and washed three times with distilled water and twice with ethanol. Finally the powders were dried at 80 ^oC under vacuum for overnight.

Cocatalysts deposition. Typically, 100 mL of deionized (DI) water, a certain amounts of catalysts and noble metal precursors ($HAuCl_4$, $RuCl_3$, Na_3RhCl_6, and H_2PtCl_6) were added into the solution. The system was evacuated several times to completely remove the dissolved air.[1] Then, it was irradiated under UV-Vis (λ > 300 nm) for 4 hours to sufficiently reduce the noble metal cations. Then, the samples were filtered and cleaned with D.I. water. Last, the solids were obtained after dried in a vacuum oven at 60 ^oC for 12 h. In comparison, Pt nanoparticles were also deposited by reduction with H_2 and $NaBH_4$. Typically, 0.2 g of g-C_3N_4 powders were immersed in 5 mL of DI water followed by ultrasonic wave bath for 5 minutes. Then, a certain amounts of H_2PtCl_6 (3 wt. %, based on Pt atoms) were added into the solution. The final resultant sample was obtained after evaporation and drying in an oven at 80 ^oC for 12 h. Thereafter, half of the sample was treated in H_2 flow at 200 ^oC for 2h. The other was treated in 0.5 M of $NaBH_4$ solution. Then the powders were finally collected after washed with DI water and dried in an oven at 60 ^oC for 12 h. PtO_x/g-C_3N_4 was synthesized by a typical immersion strategy followed by thermal treatment in the air. Typically, 0.2 g of g-C_3N_4 powders was immersed in 5 mL of DI water followed by ultrasonic wave bath for 5 minutes. Then, a certain amounts of H_2PtCl_6 (3 wt. %, based on Pt atoms) were added into the solution. The final resultant sample was obtained after evaporation and thermal treated in muffle furnace at 300 ^oC for 1 h. CoO_x/g-C_3N_4 was prepared by an in-situ photo-deposition. Typically, 0.2 g

of CN powders were dispersed in 100 mL water solution containing 1 wt. % Co^{2+}. After evacuation for several times to remove the air, it was irradiated with UV light for 4 hours. The final products were collected after filtration and drying.

Characterization methods. Powder X-ray diffraction (XRD) measurements were performed on a Bruker D8 Advance diffractometer with Cu-Kα1 radiation (λ = 1.5406 Å). Fourier transform infrared (FTIR) spectra were recorded on a BioRad FTS 6000 spectrometer, and the samples were prepared as KBr pellets. Nitrogen adsorption–desorption isotherms were collected at a Micromeritics ASAP 2020 surface area and porosity analyzer. The sample was degassed at 413 K for 5 h and then analyzed at 77 K. Transmission electron microscopy (TEM) images and high-resolution TEM (HRTEM) images were obtained using a JEOL Model JEM 2010 EX instrument at an accelerating voltage of 200 kV. Atomic force microscopy (AFM) was recorded by a Veeco Nanoscope IVa Multimode system. UV-Vis diffuse reflectance spectra (DRS) were performed on a Varian Cary 500 Scan UV-visible system. $BaSO_4$ was used as a reflectance standard. The optical properties were evaluated by F(R), and they were applied with a Kubelka-Munk correction. Photoluminescence spectra were recorded on an Edinburgh FI/FSTCSPC 920 spectrophotometer under the excitation wavelength of 400 nm at room temperature. Electrochemical measurements were conducted with a BAS Epsilon Electrochemical System in a conventional three electrode cell, using a Pt plate as the counter electrode and an Ag/AgCl electrode (3 M KCl) as the reference electrode, the active area is confined to 0.25 cm^2. The electrolyte was 0.2 M Na_2SO_4 aqueous solution without additive (pH 6.8).

First-principles calculations. The periodic DFT[2-3] calculations were implemented in the Vienna ab initio simulation package (VASP)[4-6] with the Perdew-Burke-Ernzerhof generalized gradient approximation (GGA).[7] The $C_{2s}{}^{2}{}_{p}{}^{2}$, $N_{2s}{}^{2}{}_{sp}{}^{3}$ and H_{1s} electrons were treated as valence electrons. The interactions between the cores and valence electrons were described by the projected augmented wave (PAW)[8] method and the plane-wave cutoff energy was set as 550 eV. The single layer structures of melon were used in the calculations. The k-point was sampled with a Monkhorst-Pack grid of 5×5×1 mesh.[9] The atomic coordinates and lattice parameters were fully relaxed until the total energies were converged to 10^{-5} eV and the Hellmann–Feynman forces on each atom were less than 0.01 eV Å.

Photocatalytic Test. Reactions were carried in a Pyrex top-irradiation reaction vessel connected to a glass closed gas system. Water splitting reaction was performed by dispersing 200 mg of catalyst powders in an aqueous solution (100 mL) without any sacrificial agent. 3 wt. % Pt was loaded on the surface of catalysts by *in situ* photo-deposition method using H_2PtCl_6. The reaction solution was evacuated several times to remove air completely prior to irradiation with a 300 W Xeon lamp with a working current of 15 A (Shenzhen ShengKang Technology Co., Ltd, China, LX300F). The wavelength of the incident light was controlled by applying some appropriate long-pass cut-off filters. The temperature of the reaction solution was maintained at room temperature by a flow of cooling water during the reaction. The evolved gases were analyzed by gas chromatography equipped with a thermal conductive detector (TCD) and a 5Å molecular sieve column, using argon as the carrier gas.

The apparent quantum yield (AQY) for H_2 evolution was similar to that for water splitting reaction. The irradiated light was controlled by a band-pass filter 405 nm. The irradiation area was controlled as 6 cm^2. The total intensity of incident light irradiation was measured as 2.18 mW cm^{-2} for 405 nm

(the irradiation intensity was determined by ILT 950 spectroradiometer). The AQY was calculated as follow:

$$\text{AQY}= \frac{N_e}{N_p} \times 100\% = \frac{2 \times M \times N_A \times h \times c}{S \times P \times t \times \lambda} \times 100\%$$

Where, N_p is the total incident photons, N_e is the total reactive electrons, M is the amount of H_2 molecules, N_A is Avogadro constant, h is the Planck constant, c is the speed of light, S is the irradiation area, P is the intensity of irradiation light, t is the photoreaction time, λ is the wavelength of the monochromatic light.

Reference:

1. Maeda K. *et al.* Photocatalytic activities of graphitic carbon nitride powder for water reduction and oxidation under visible light. *J. Phys. Chem. C* **113***,* 4940-4947 (2009).
2. Hohenberg P. & Kohn W. Inhomogeneous electron gas. *Phys. Rev.* **136**, B864-B871 (1964).
3. Kohn W. & Sham L. J. Self-consistent equations including exchange and correlation effects. *Phys. Rev.* **140**, A1133-A1138 (1965).
4. Kresse G. & Furthmüller J. Efficiency of ab-initio total energy calculations for metals and semiconductors using a plane-wave basis set. *Comput. Mater. Sci.* **6**, 15-50 (1996).
5. Kresse G. & Furthmüller J. Efficient iterative schemes for *ab initio* total-energy calculations using a plane-wave basis set. *Phys. Rev. B* **54**, 11169-11186 (1996).
6. Kresse G. & Joubert D. From ultrasoft pseudopotentials to the projector augmented-wave method. *Phys. Rev. B* **59**, 1758-1775 (1999).
7. Perdew J. P., Burke K. & Ernzerhof M. Generalized gradient approximation made simple. *Phys. Rev. Lett.***77**, 3865-3868 (1996).
8. Blöchl P. E. Projector augmented-wave method. *Phys. Rev. B* **50**, 17953-17979 (1994).
9. Monkhorst H. J. & Pack J. D. Special points for Brillouin-zone integrations. *Phys. Rev. B* **13**, 5188-5192 (1976).

Table S1. Physicochemical properties of different precursors derived g-C_3N_4 samples

Entry	Catalysts	S_{BET} / m^2 g^{-1}	Band gap / eV	H_2 evolution rate /μmol h^{-1}	O_2 evolution rate /μmol h^{-1}	Water splitting rate / μmol h^{-1}	
						H_2	O_2
1	CNU	61	2. 8	60	5	8.5	3.2
2	CND	8	2.7	12.9	0.7	0.2	-
3	CNA	9	2.71	21.9	0.8	0.3	-
4	mpg-CN	67	2.73	149	0.83	0.4	-

Entry 1-3 is corresponded to urea, DCDA and ATC-derived g-C_3N_4 samples; entry 4 is mesoporous g-C_3N_4 (mpg-C_3N_4) prepared via hard-template strategy. Surface area was determined by the nitrogen adsorption–desorption isotherms; band gap was calcu.lated by the diffuse reflection spectra; H_2 and O_2 evolution rates were performed under $\lambda > 420$ nm and $\lambda > 300$ nm, respectively; water splitting rate was carried out under $\lambda > 300$ nm, 3 wt. % Pt was *in-situ* deposited as cocatalyst.

Table S2. The binding energies and relative intensities of different platinum species observed from the Pt_{4f} X-ray photoelectron spectra of Pt/g-C_3N_4 samples.

Entry	Pt contents (%)	Chemical valence of Pt	Binding energies of $4f_{7/2}$ (eV)	FWHM fit param (eV)	Relative intensity (%)	Pt/(Pt^{2+}+Pt^{4+})
		0	72.4	2.15	82	
1	0.2 wt.	+2	74.3	0.48	18	4.54:1
		+4	-	-	-	
		0	72.1	2.17	62	
2	1 wt.	+2	74.4	1.55	30	1.61:1
		+4	76.6	1.48	8	
		0	72.2	2.35	60	
3	3 wt.	+2	74.5	1.58	36	1.45:1
		+4	76.3	1.35	4	
		0	72.3	2.44	53	
4	5 wt.	+2	74.7	1.60	41	1.15:1
		+4	76.3	1.38	5	

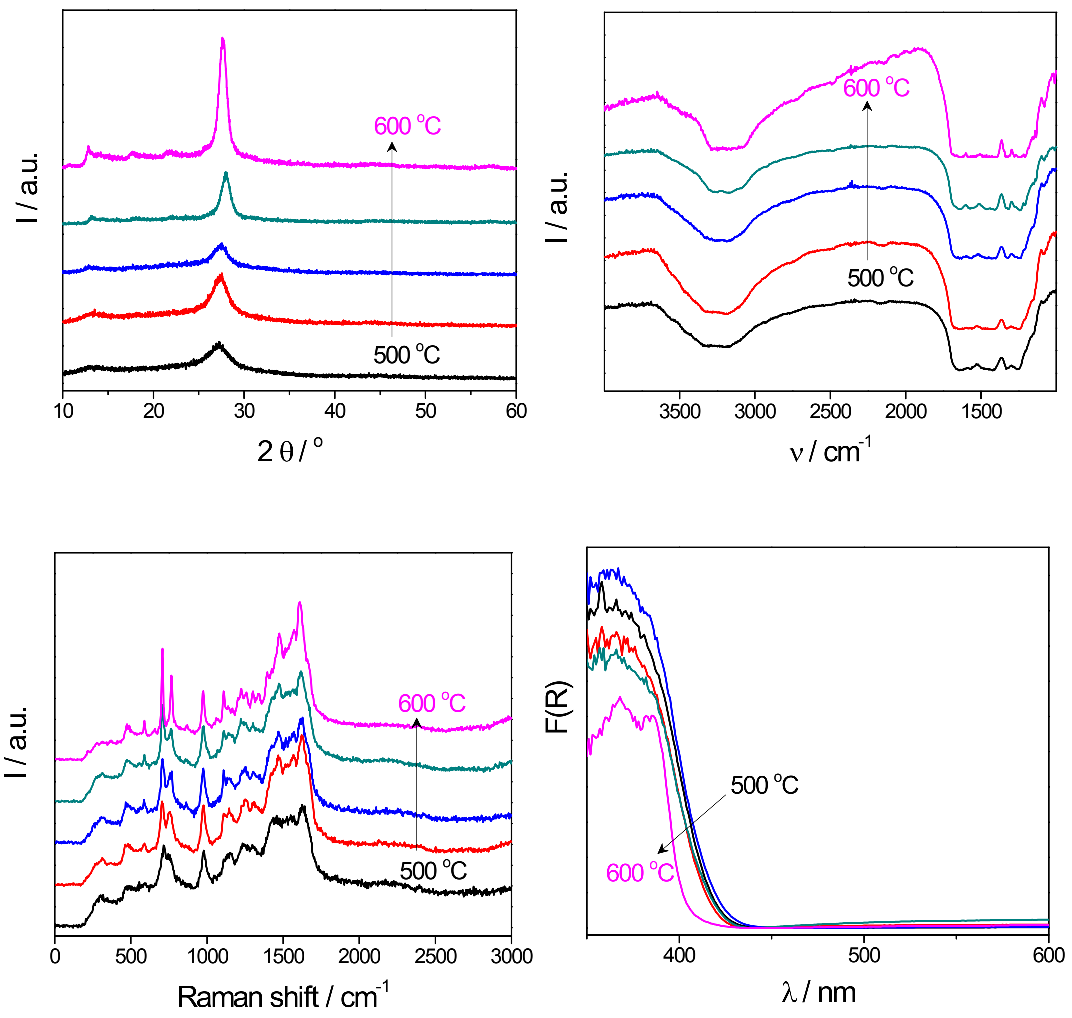

Figure S1. (a) Powder XRD patterns, (b) FT-IR variations, (c) Raman spectra and (d) UV-vis DRS spectra of g-C_3N_4 at different temperatures (500, 525, 550, 575, 600 °C).

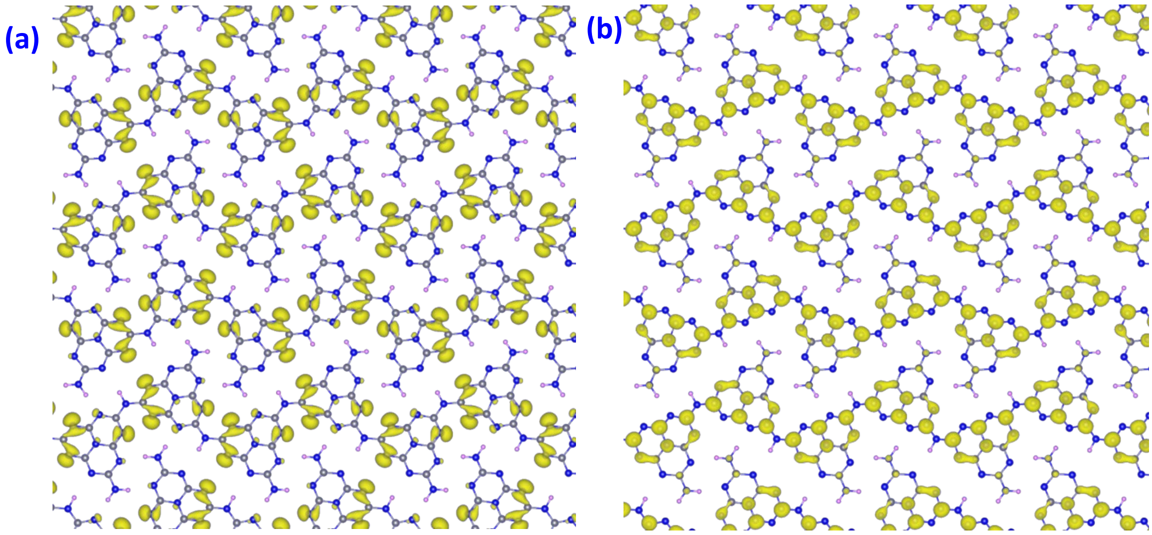

Figure S2. Band decomposed charge density of (a) valence band maximum and (b**)** conduction band minimum.

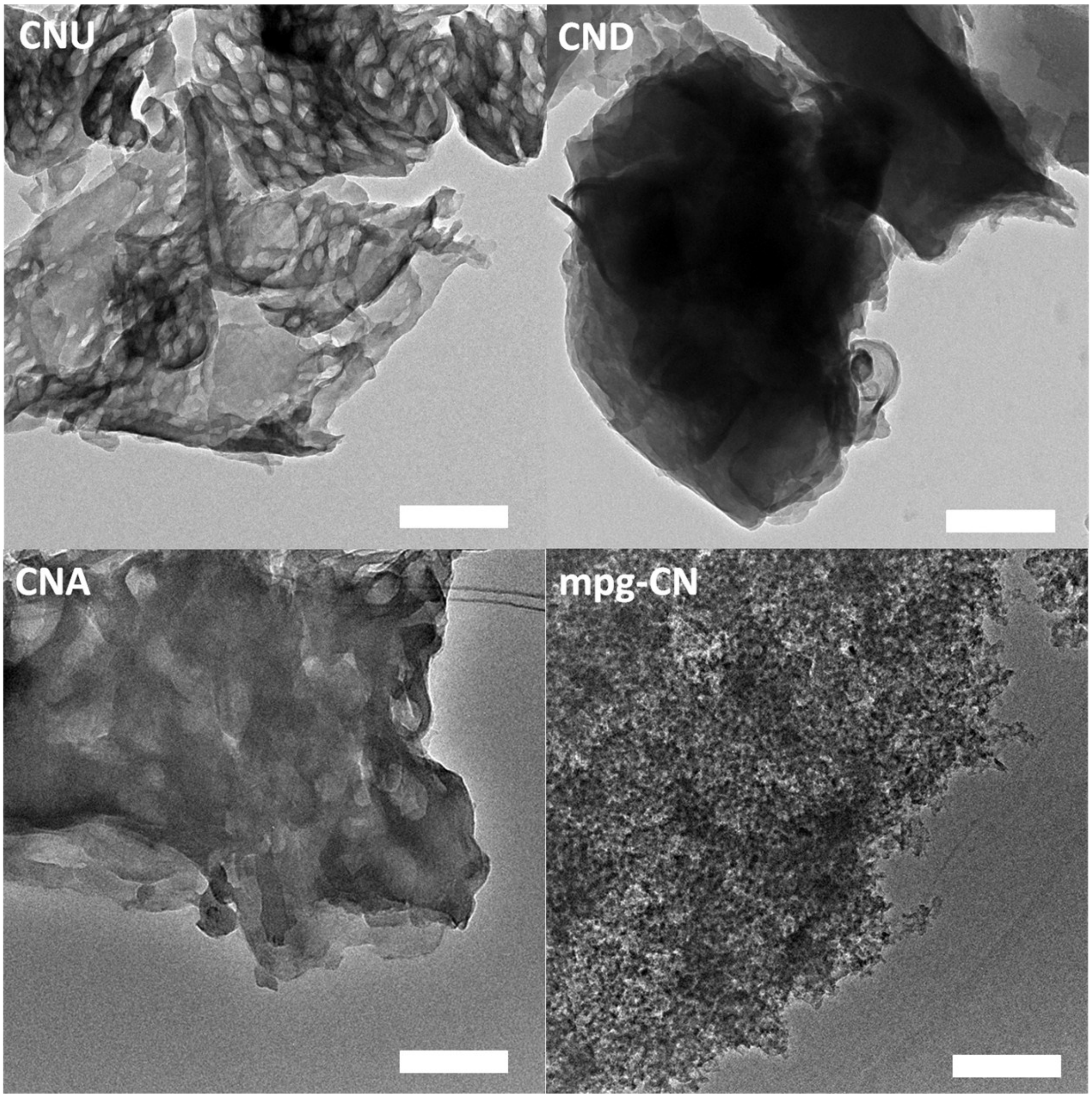

Figure S3. Transmission electron microscopy (TEM) imagines of different precursors derived g-C_3N_4 samples. (CNU: urea, CND: Dicyandiamide, CNA: ammonium thiocyanate; mpg-CN: mesoporous g-C_3N_4) Scale bar is 200 nm.

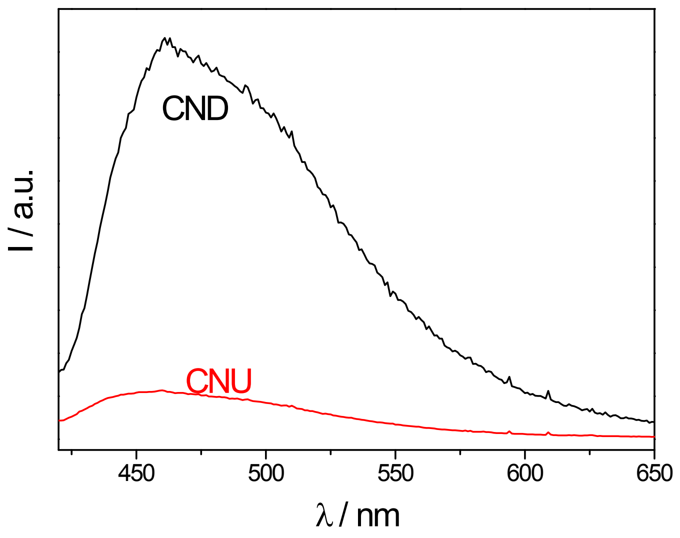

Figure S4. Photoluminescence spectra (PL) of different precursors (CNU: urea, CND: Dicyandiamide) derived g-C_3N_4 samples.

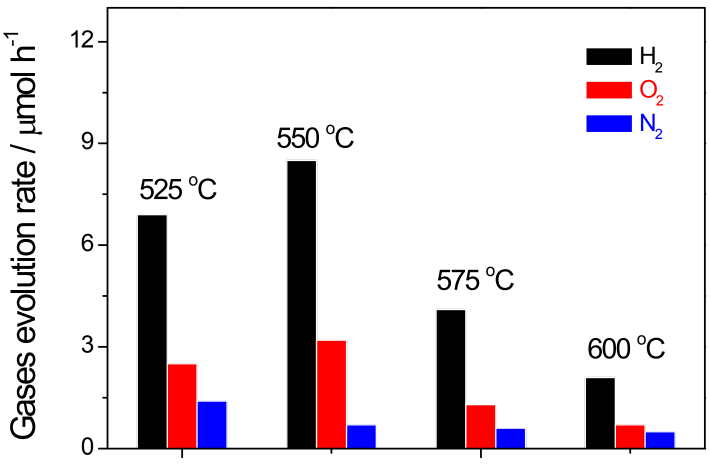

Figure S5. Water splitting rate of 3 wt. % Pt loaded g-C_3N_4 prepared at different temperatures (525, 550, 575, 600 °C) samples (0.2 g) under λ>300 nm.

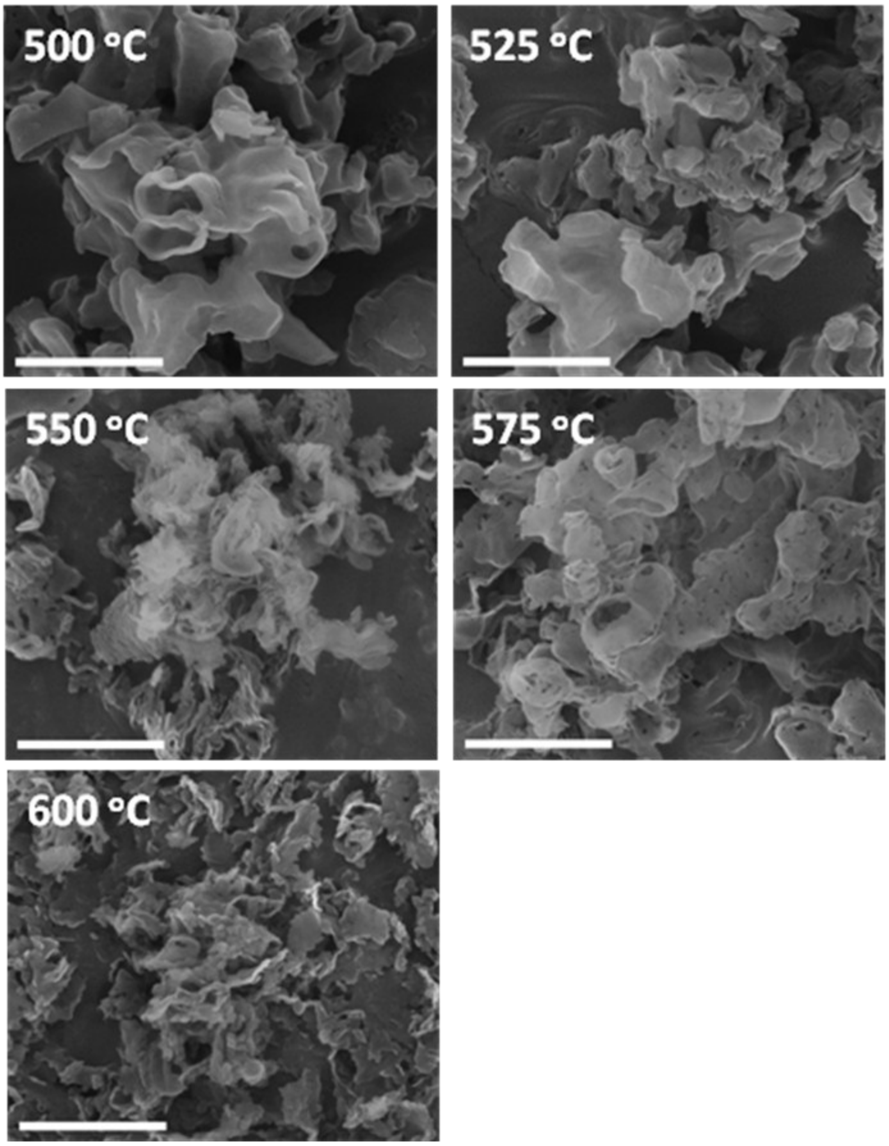

Figure S6. SEM pictures of urea-derived g-C_3N_4 prepared at different temperatures (500, 525, 550, 575, 600 ^oC).

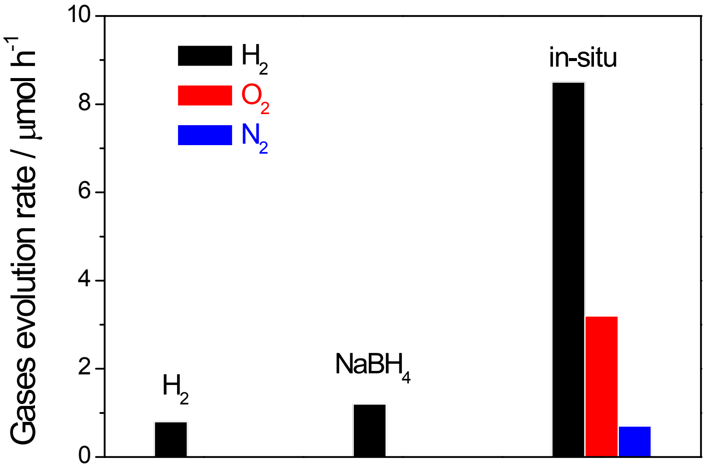

Figure S7. Water splitting rate of different reduction methods for 3 wt. % Pt loaded g-C_3N_4 sample under λ>300 nm. **a:** reduced by H_2 flow at 200 ^oC, **b:** reduced in 0.5 M $NaBH_4$ solution, **c:** in-situ photodeposited.

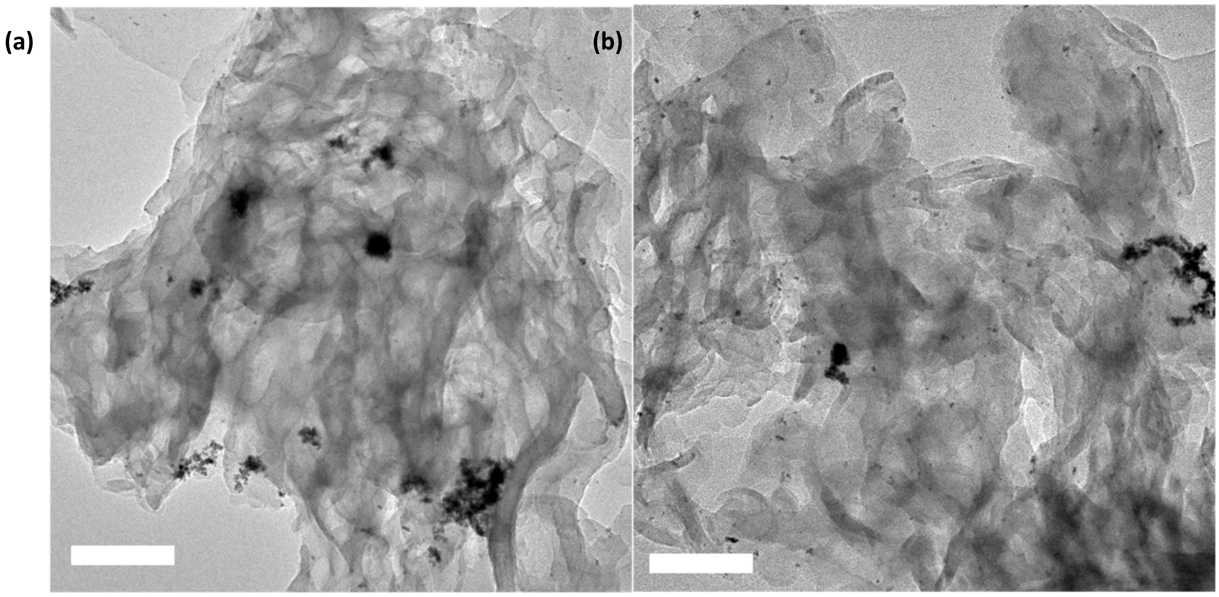

Figure S8. Transmission electron microscopy (TEM) imagines of 3 wt. % Pt loaded g-C_3N_4 samples. Prepared by reduction in 0.5 M $NaBH_4$ (a) and H_2 flow (b), Scale bar is 100 nm.

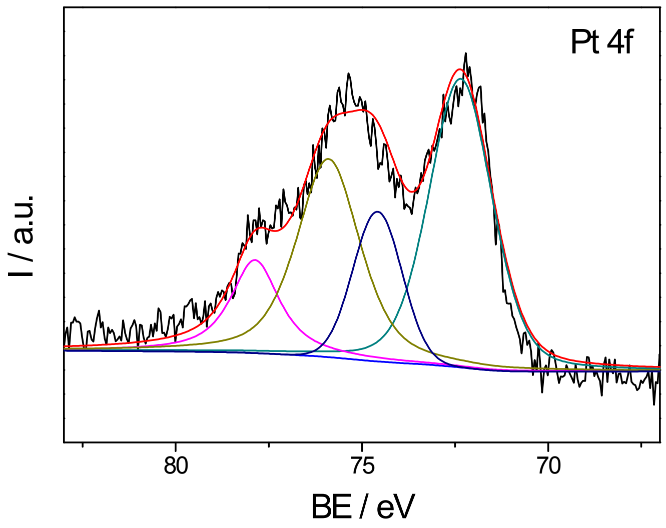

Figure S9. High-resolution X-ray photoelectron spectra of 3 wt % Pt loaded g-C_3N_4 sample reduced in 0.5 M $NaBH_4$ solution.

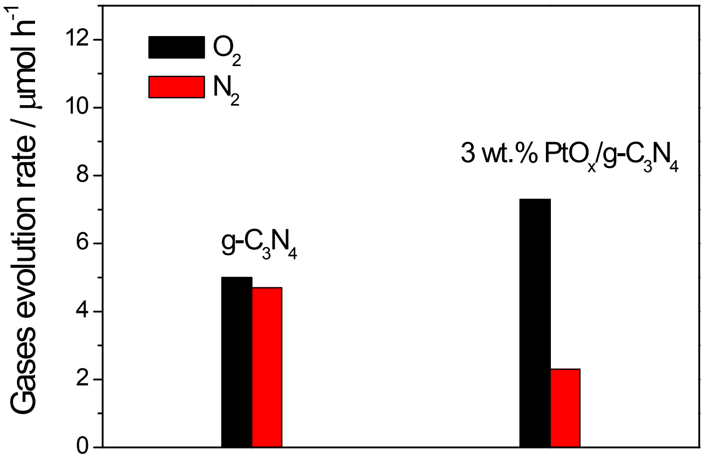

Figure S10. Water oxidation rate of pure and 3 wt. % PtO_x loaded g-C_3N_4 samples under λ>300 nm. Conditions: 50 mg of catalyst, 0.1 M of $AgNO_3$ as electron accepter, 0.2 g of La_2O_3 as pH buffer.

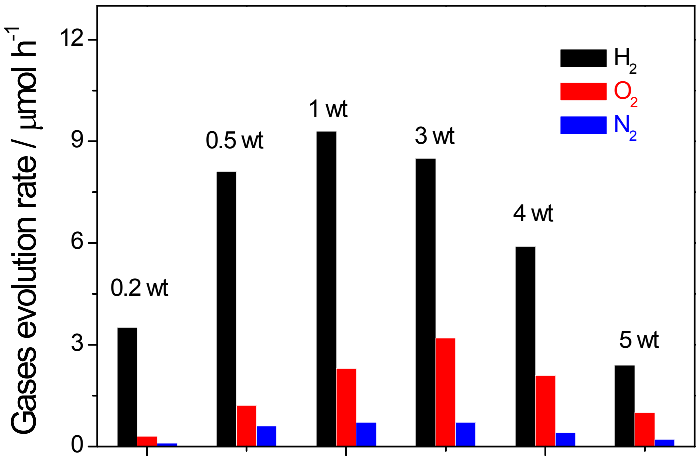

Figure S11. Water splitting rate of different amounts (0.2, 0.5, 1, 3, 4, 5 wt. %) of Pt loaded g-C_3N_4 (0.2 g) samples under λ>300 nm.

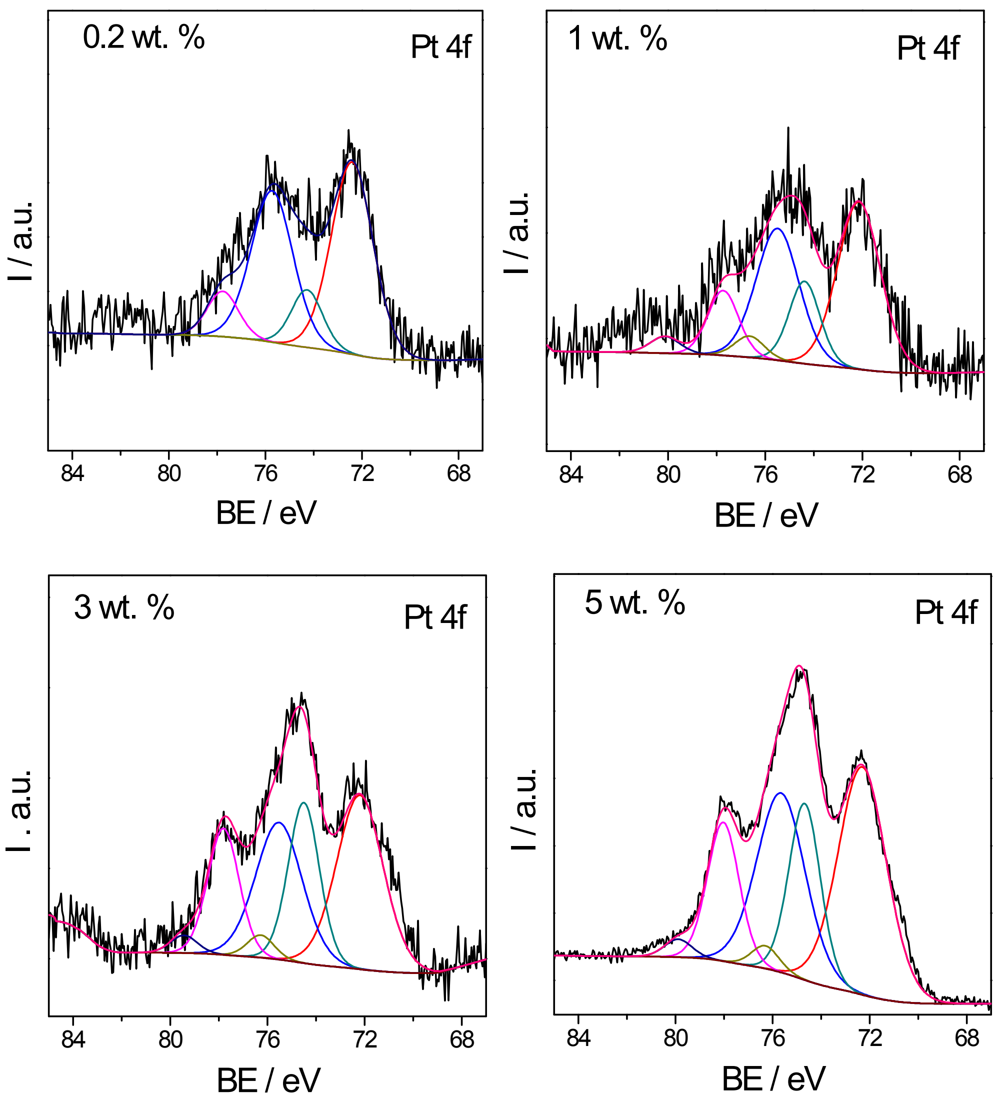

Figure S12. High-resolution XPS analysis of 0.2 wt., 1 wt., 3 wt., and 5 wt. % Pt loaded g-C_3N_4 samples.

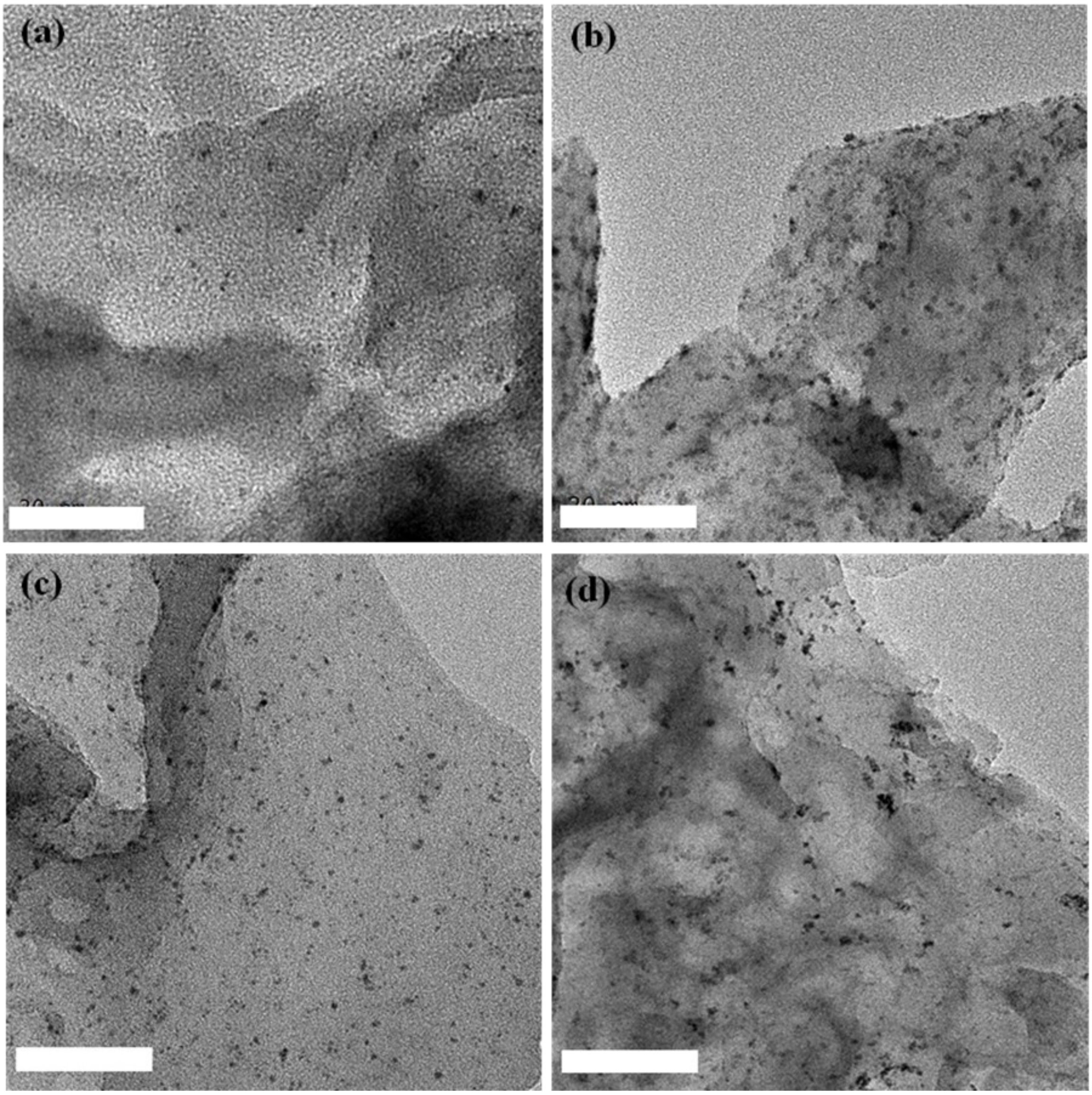

Figure S13. Transmission electron microscopy (TEM) imagines of (a) 0.2, (b) 1, (c) 3 and (d) 5 wt. % Pt loaded g-C_3N_4 samples. Scale bar is 50 nm.

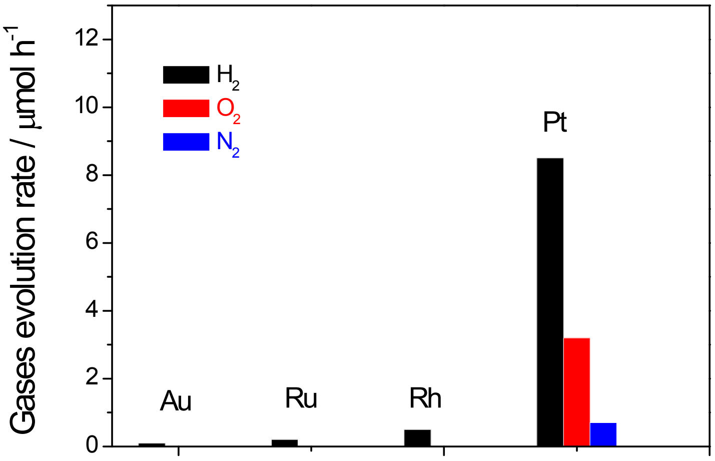

Figure S14. Water splitting rates of different noble metals (3 wt. % Au, Ru, Rh, Pt) *in-situ* photo-deposited g-C_3N_4 samples under $\lambda > 300$ nm.

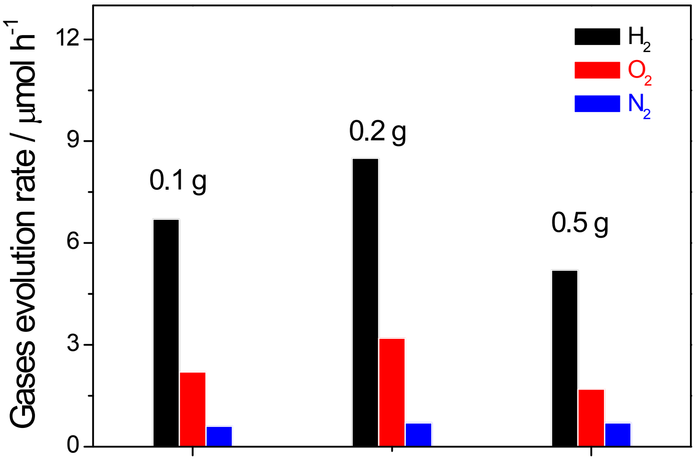

Figure S15. Water splitting rate of 3 wt. % Pt loaded with different amounts of g-C_3N_4 samples (0.1, 0.2, 0.5 g) under λ>300 nm.

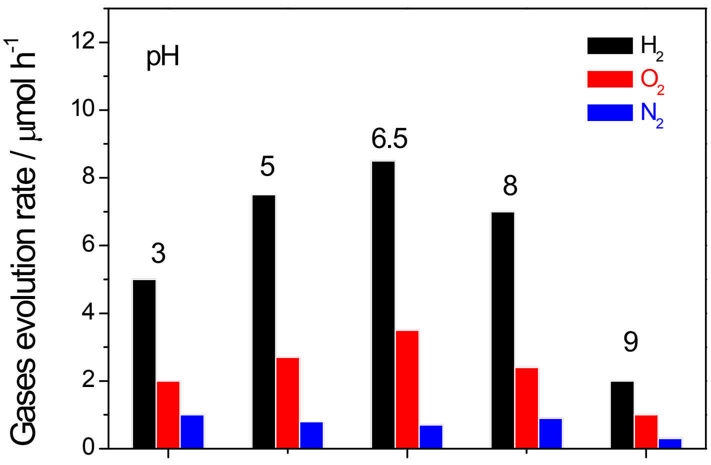

Figure S16. pH value dependence of water splitting rate of 3 wt. % Pt loaded g-C_3N_4 samples under λ>300 nm. The pH value of the solution was controlled by 1 M HCl or 1 M NaOH.

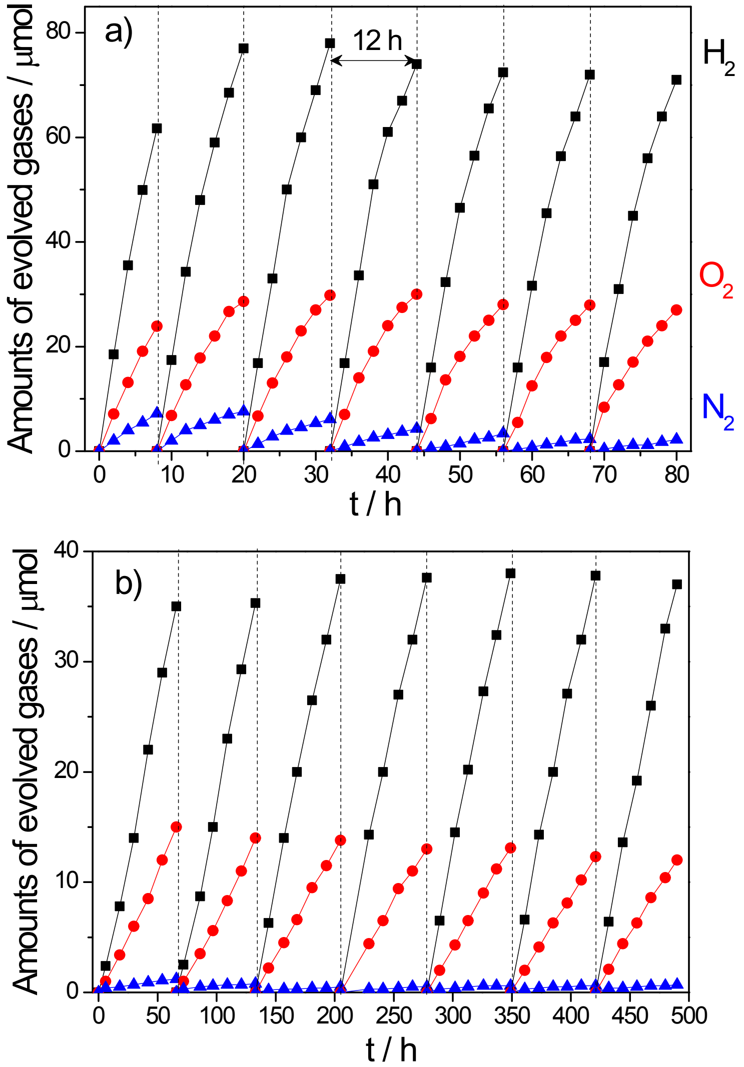

Figure S17. Time course water splitting activities of 3 wt. % Pt modified g-C_3N_4 nanosheet under (a) UV-Vis (λ > 300 nm) and visible light irradiation (λ > 420 nm).

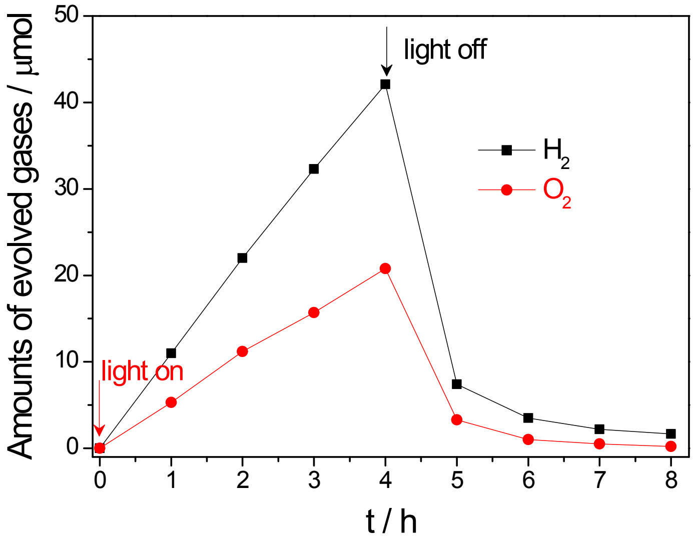

Figure S18. Overall water splitting of 3 wt. % Pt and PtO_x modified g-C_3N_4 photocatalysts under UV light irradiation (λ > 300 nm) and in dark.

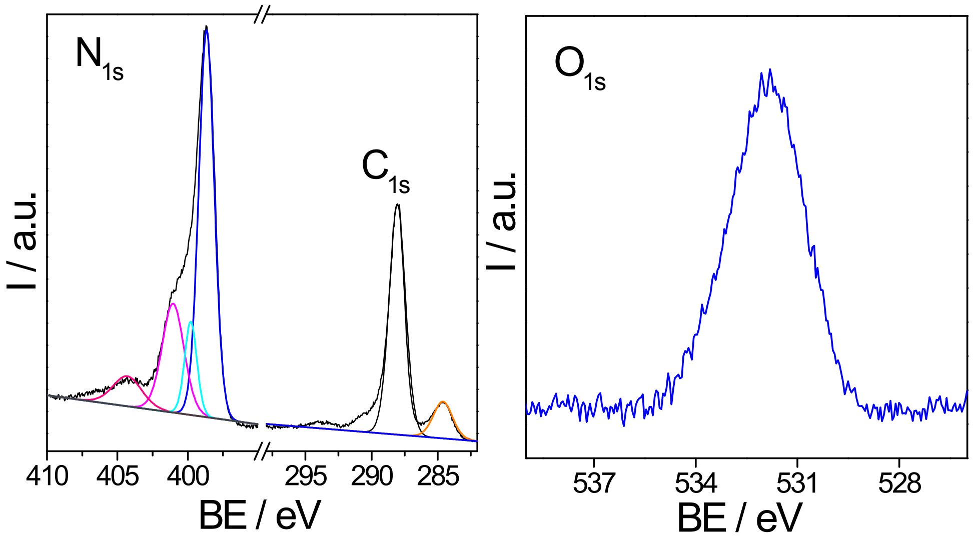

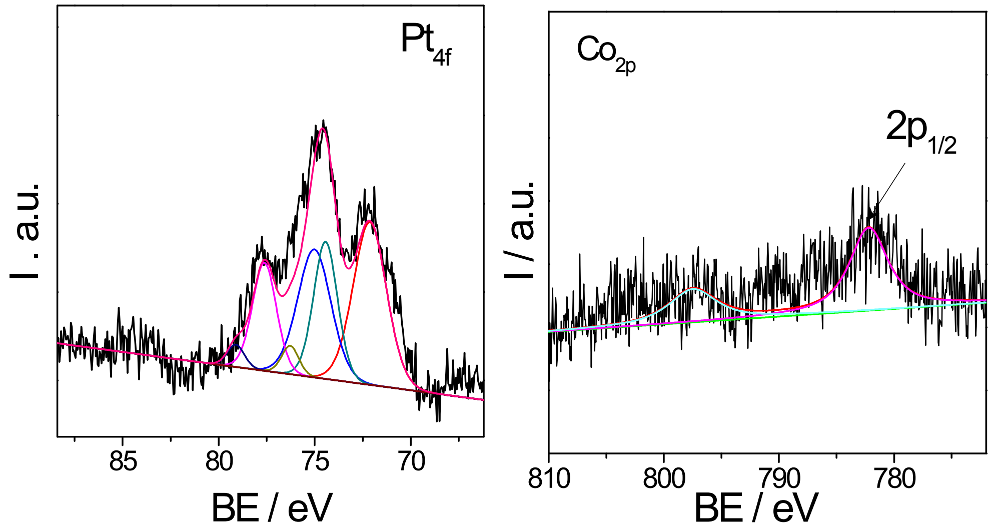

Figure S19. High-resolution XPS analysis of C_{1s}, N_{1s}, O_{1s}, Pt_{4f}, and Co_{2p} of 3 wt. % Pt, PtO_x and 1 wt. % CoO_x modified g-C_3N_4 polymers.

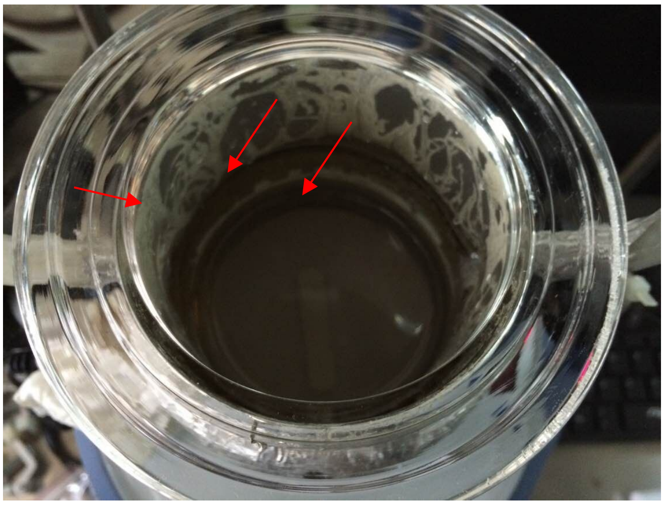

Figure S20. Digital photograph of samples were stacked onto the inner side of the glass reaction vessel.

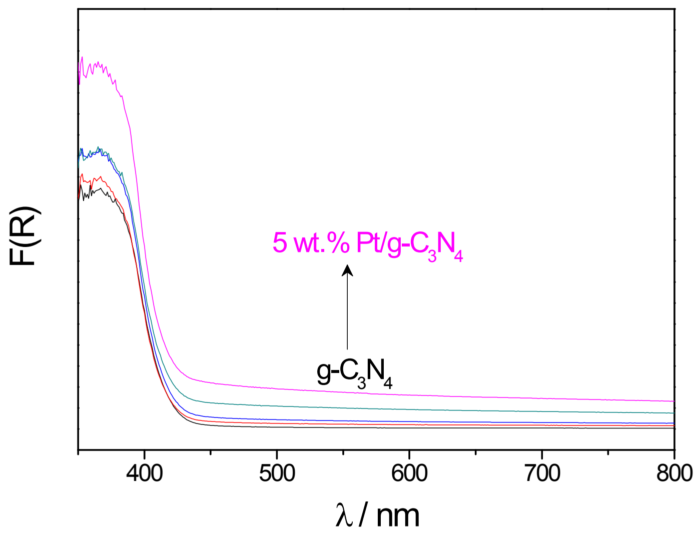

Figure S21. UV-Vis diffuse reflection spectra (DRS) of 0, 0.2, 1, 3 and 5 wt. % Pt loaded g-C_3N_4 samples.

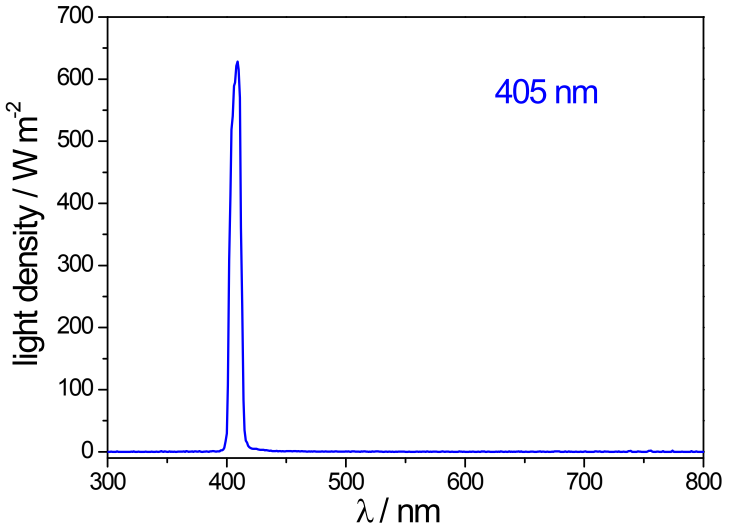

Figure S22. The light irradiation spectra of the xenon lamp (300 W) with a 405 nm band-pass filter. Irradiance intensity was determined as 5.2 mW cm^{-2}.

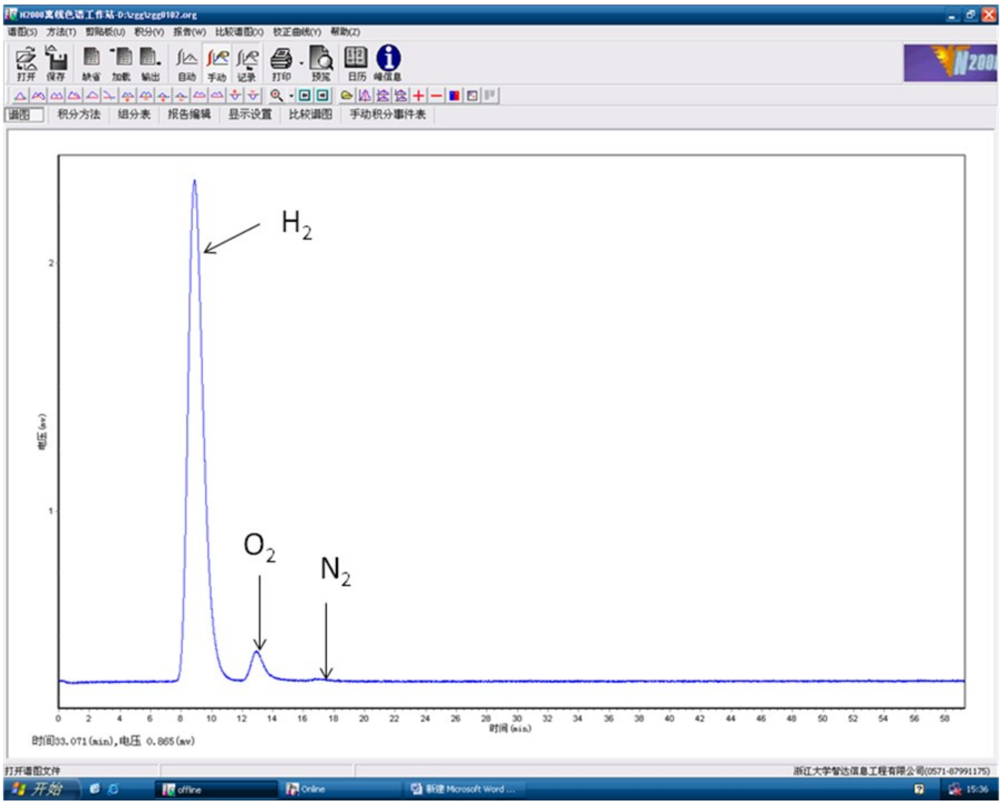

Figure S23. An original water splitting chromatogram of the modified g-C_3N_4 samples, which is monitored by the on-line gas chromatogram to quantify the H_2, O_2 and N_2 gases produced.
